# Cytokines as potential biomarkers for the diagnosis of *Mycobacterium bovis* infection in Mediterranean buffaloes (*Bubalus bubalis*)

**DOI:** 10.3389/fvets.2024.1512571

**Published:** 2024-12-24

**Authors:** Giulia Franzoni, Federica Signorelli, Piera Mazzone, Anna Donniacuo, Giovanna De Matteis, Francesco Grandoni, Lorena Schiavo, Susanna Zinellu, Silvia Dei Giudici, Javier Bezos, Esterina De Carlo, Giorgio Galiero, Francesco Napolitano, Alessandra Martucciello

**Affiliations:** ^1^Department of Animal Health, Istituto Zooprofilattico Sperimentale della Sardegna, Sassari, Italy; ^2^CREA-Consiglio per la Ricerca in Agricoltura e l’Analisi dell’Economia Agraria, Centro di ricerca Zootecnia e Acquacoltura (Research Centre for Animal Production and Aquaculture), Monterotondo (RM), Italy; ^3^Istituto Zooprofilattico Sperimentale dell’Umbria e delle Marche “Togo Rosati”, Perugia, Italy; ^4^National Reference Centre for Hygiene and Technologies of Mediterranean Buffalo Farming and Productions, Istituto Zooprofilattico Sperimentale del Mezzogiorno, Salerno, Italy; ^5^VISAVET Health Surveillance Centre, Complutense University of Madrid, Madrid, Spain; ^6^Facultad de Veterinaria, Departamento de Sanidad Animal, Universidad Complutense de Madrid, Madrid, Spain

**Keywords:** *Mycobacterium bovis*, biomarkers, interleukins, chemokines, Mediterranean buffaloes

## Abstract

*Mycobacterium bovis* (*M. bovis*) is the primary agent of bovine tuberculosis (TB) in Mediterranean buffalo, which has a negative economic impact on buffalo herds. Improving TB diagnostic performance in this species represents a key step to eradicate efficiently this disease. We have recently shown the utility of the IFN-γ assay in the diagnosis of *M. bovis* infection in Mediterranean buffaloes (*Bubalus bubalis*), but other cytokines might be useful immunological biomarkers of this infection. We therefore investigated the utility of key immune cytokines as diagnostic biomarkers of *M. bovis* infection in this species. Thirty-six Italian Mediterranean buffaloes were used in this study: healthy animals (*N* = 11), infected (IFN-γ test positive, no post-mortem lesions, no *M. bovis* detection; *N* = 14), and affected (IFN-γ test positive, visible post-mortem lesions; *N* = 11). Heparin blood samples were stimulated with bovine purified protein derivative (PPD-B), alongside controls, and 18–24 h later plasma were collected. Levels of 14 key cytokines were measured: IFN-γ, IL-17, IL-4, IL-10, TNF, IL-1α, IL-1β, IL-6, IP-10, MIP-1α, MIP-1β, MCP-1, IL-36Ra, and VEGF-A. We observed that both infected and affected animals released higher levels of IFN-γ, IL-17, IL-10, TNF, IL-1α, IL-6, MIP-1β, in response to PPD-B compared to healthy subjects. *Mycobacterium bovis* infected animals released also higher levels of IL-1β and IP-10 in response to PPD-B compared to healthy subjects, whereas only tendencies were detected in affected animals. Affected animals only released MIP-1α in response to PPD-B compared to healthy subjects and in this group higher values of PPD-B specific TNF was also observed. Finally, canonical discriminant analysis (CDA) was used to generate predictive cytokine profiles by groups. Our data suggest that IL-10, TNF, IL-1α, IL-6, MIP-1β could be useful biomarkers of TB in Mediterranean Buffalo and can improve the TB diagnostic performance in this specie.

## 1 Introduction

Bovine tuberculosis (TB) is a zoonotic disease caused by mycobacteria of the *Mycobacterium tuberculosis* complex (MTBC), mainly *M. bovis*, in bovines (including all *Bos* species, and *Bubalus bubalus*) (1, 2).

Tuberculosis is a chronic, progressive disease, which most commonly affects the lung (1). Following initial infection, there is often an asymptomatic latent period, and the progress of the infection depends on the interplay between the pathogen and host defence mechanisms. One of the main features of the disease is the formation of typical tuberculous granuloma (inflammatory mononuclear cell infiltrates), which limits the spread of mycobacteria into the host (1).

Like other members of the family *Bovidae*s, Mediterranean buffalo (*Bubalus bubalis*) is susceptible to *M. bovis* infection. In Mediterranean buffalo TB represents not only a threat to human health, but also has a negative economic impact on buffalo herds. In the past years, TB prevalence in buffaloes increased in many countries, such as India and Brazil (3, 4), and the disease is still present in the European Union, despite several efforts to eradicate it. In Italy, TB in Mediterranean buffalo is mainly present in the Campania region (South of Italy), where more than 70% of the buffalo stocks are bred. In that area, several TB outbreaks occurred in buffalo stocks in the last decade (5).

The TB eradication program is based mainly on slaughterhouse surveillance and ante mortem tests, such intradermal tuberculin test (IDT) and interferon-gamma (IFN-γ) test. However, IDT presents some disadvantages in buffalo, such as low specificity (due mainly to sensitization with non-tuberculosis mycobacteria) and difficulties in test execution and interpretation (because of characteristics of buffalo skin) (6–8).

The IFN-γ test has been widely used in the diagnosis of *M. bovis* infection in cattle and we have recently shown the utility of this test in Mediterranean buffaloes (5, 6). With the implementation of the European “Animal Health law” (Regulation (EU) 2016/429 (9), Regulation (EU) 2020/689 (10)), the IFN-γ test has become an official test approved for use in buffalo. IFN-γ is a cytokine frequently used to assess cell-mediated immune response to intracellular pathogens (11) and *M. bovis* infection is characterized by a cell-mediated immune response (CMI) that precedes humoral responses (1). In infected animals, this cytokine is released *in vitro* by sensitized T cells after Purified Protein Derivative (PPD) antigens stimulation (12, 13). Not only IFN-γ, but also other interleukins, chemokines or cytokines are involved in the cell-mediated immune response to *M. bovis* (13) and they can be considered potential immunological biomarkers of this infection. In addition, a combination of host cytokine biomarkers may increase TB diagnostic performance. Previous studies reported indeed that the parallel measurement of IFN-γ and the interferon gamma-inducible protein 10 (IP-10) improved the detection of *M. bovis* infection in cattle (14) and African buffaloes (15, 16).

Improving TB diagnostic performance in this specie represents a key step to identify the highest number of infected animals present in a herd and to eradicate rapidly TB from infected area. In our study, we therefore investigated for the first time the utility of 14 key immune cytokines as diagnostic biomarkers of *M. bovis* infection in Mediterranean buffaloes.

## 2 Materials and methods

### 2.1 Ethical statements

Mediterranean buffaloes used in this study were analyzed within the context of the official TB eradication program in accordance with European and Italian legislation [Regulation (EU) 2016/429 (9), Regulation (EU) 2020/689 (10), O.M. 28/05/2015 and subsequent amendments (17)] and regional regulations [DGRC 104/2022 and subsequent amendments (18)], therefore were not considered experimental animals.

### 2.2 Animals and study design

Thirty-six Italian Mediterranean buffaloes were enrolled in the present study.

*Mycobacterium bovis* exposed animals were selected from herds with confirmed TB outbreaks. According to current legislation (see 2.1), TB infection status was determined by ante-mortem tests, such as the single intradermal tuberculin test (SIT) and the IFN-γ test. IFN-γ test was carried out by Istituto Zooprofilattico Sperimentale del Mezzogiorno (IZSME) Italy (see 2.3).

SIT and IFN-γ test positive reactors were slaughtered in accordance with national and regional legislation and then the presence of TB-like lesions was evaluated. The organs were sent to the IZSME for investigation of the presence of *M. bovis* using PCR for *M. bovis* DNA detection and culture isolation of MTBC mycobacteria, according to the World Organization for Animal Health (WOAH) Terrestrial Manual protocols (19) (IZSME, Italy) (See 2.4).

Infected animals belong to herds with TB outbreaks, where *M. bovis* was isolated from at least one animal. Uninfected animals were selected instead from Officially Tuberculosis-Free (OTF) herds in the Campania region (Southern Italy). These animals tested negative during the SIT or IFN-γ screening tests performed in the last 6 years. Animals were divided into three groups based on results of IFN-γ test and post-mortem examinations (see 2.3, 2.4): healthy uninfected (from OTF herds, IFN-γ test negative; *N* = 11), infected (IFN-γ test positive, and post-mortem examinations negative: no TB-like lesions, PCR or culture negative; *N* = 14), and affected (IFN-γ test positive, visible TB-like lesions, PCR or culture positive; *N* = 11).

### 2.3 Whole blood stimulation and IFN-γ test

Blood samples were collected from the jugular vein in lithium-heparin vacutainer tubes (BD Biosciences) and transported to the laboratory within 8 h from collection.

Heparin blood samples of each animal were dispensed in aliquots of 1 mL and stimulated with Phosphate-buffered saline (PBS, Nil Control Antigen), 10 μg of PPD-B (BOVIGAM™ Thermo-Fisher Scientific, Schlieren, Switzerland), and Pokeweed Mitogen (PWM, final concentration 1 μg/mL, control of lymphocyte reactivity), respectively. Then, samples were incubated for 18–24 h at 37°C in a humidified atmosphere and then plasma were collected. A first aliquot was used to determine the IFN-γ levels using the BOVIGAM™, whereas a second aliquot was stored at −80°C for evaluation of cytokine levels with multiplex ELISA (see 2.5).

IFN-γ levels were determined within the context of the TB control program, using the BOVIGAM™ sandwich ELISA test, according to the manufacturer’s instructions (Life Technologies, Thermo-Fisher Scientific). Samples were considered positive when the differences between PPD-B and PBS was ≥0.1 OD according to the European Standard Operating Procedures [SOP/004/EURL (20)] of the European Union Reference Laboratory for Bovine Tuberculosis (EURL-TB).

### 2.4 Post-mortem diagnostic tests

All the *M. bovis* exposed buffaloes under study (IFN-γ test positive, *N* = 25) were slaughtered according to current legislation. Official veterinarians executed post-mortem examinations to detect the presence of typical TB lesions. Several tissue samples (tonsils, retropharyngeal, mandibular, tracheobronchial, mediastinal, mesenteric, hepatic, sub-iliac, supra-mammary, popliteal, prescapular lymph nodes, lung, liver, and spleen) were collected and transported to the laboratory. A tissue direct PCR was carried out to detect *M. bovis* DNA from organs, and a culture examination for MTBC mycobacteria isolation, all diagnostics procedures were performed in accordance to the WOAH Terrestrial Manual protocols (19, 21, 22). From culture, identification of mycobacteria strains isolated was performed using molecular methods previously described by Boniotti et al. (23). In details, the gyrB gene analysis by restriction fragment length polymorphism (RFLP) assay was performed for differentiation among *M. bovis*, *M. caprae*, *M. microti*, and human adapted MTBC species (23).

### 2.5 Evaluation of plasma cytokines after stimulations

Levels of IFN-γ, IL-1α, IL-1β, IL-4, IL-6, IL-10, IL17, MIP-1α, MIP-1β, IL-36Ra, IP-10, MCP-1, TNF, VEGF-A in plasma were measured using Bovine Cytokine/Chemokine Magnetic Bead Panel Multiplex assay (Merck Millipore, Darmstadt, Germany) and a Bioplex MAGPIX Multiplex Reader (Bio-Rad, Hercules, CA, USA), according to the manufacturers’ instructions (24). All samples were tested in duplicate (two technical replicates).

The levels of *M. bovis* specific cytokine responses in plasma were determined by subtracting baseline cytokines concentrations (PBS, nil control) from the concentration of cytokines measured in the PPD-B condition.

### 2.6 Statistical analysis

Levels of the 14 tested cytokines/chemokines were analyzed using the general linear model (GLM) to estimate the mean of each trait per stimulus (PBS, PPD-B, and PWM) within groups (healthy, infected, and affected):


$$Y_{jk}=\mu +G_{j}+e_{jk}$$

where Y_jk_ is the trait measured for each animal, μ is the overall mean, G_j_ is the fixed effect of the stimuli (j = 3 levels: PBS, PPD-B, and PWM), and e_jk_ is the random residual effect of each observation.

The statistical significance of all traits and least-square means were determined by Dunnet’s multiple test available in the GLM procedure.

Furthermore, the difference (∆_cytokine) between the level of each specific cytokine measured in the bovine purified protein derivative condition (PPD-B) and its baseline cytokine concentration (PBS) were analyzed by Tukey multiple comparison test and were displayed with GraphPad Prism 10.01 (GraphPad Software Inc., La Jolla, CA, USA).

A multivariate approach was conducted using canonical discriminant analysis (CDA) on 11 ∆_cytokines by the CANDISC Procedure. ∆_IL4, ∆_IL-36a, and ∆_MCP-1 were removed because they did not show significant differences among the three groups.

The significance level for both statistical analyses was set at a *p* < 0.05.

The CDA was conducted categorizing animals prior into healthy, infected, and affected. The CANDISC method was utilized to estimate linear functions of all quantitative variables that best discriminated the groups while minimizing the variation within each group. All statistical analyses were performed with SAS software version 9.4.

## 3 Results

Thirty-six Italian Mediterranean buffaloes, divided into healthy, infected, and affected were used in this study. Heparin blood samples were stimulated for 18–24 h with PPD-B, alongside the nil control antigen (PBS) and a control of lymphocyte reactivity (PWM).

In all tested animals, on plasma collected 18–24 h after the stimulation phase of IFN-γ test, higher release of IFN-γ, IL-17, IL-4, IL-10, TNF, and IP-10 was detected in response to PWM stimulation compared to PBS, indicating that lymphocyte reactivity was not altered (Table 1).

**Table 1:** Production of cytokines in whole blood from healthy, infected and affected Mediterranean buffaloes.

	PBS	PPD-B	PWM	PBS *vs* PPD-B	PBS *vs* PWM
	LSM ± SEE	LSM ± SEE	LSM ± SEE	Dunnet multiple test (*p*-value)	
HEALTHY					
Cytokines					
IFN-γ	2 ± 82	25 ± 82	832 ± 82	0.9705	**0.0001**
IL-17	2 ± 34	15 ± 34	308 ± 34	0.9478	**0.0001**
IL-4	81 ± 22	90 ± 22	179 ± 22	0.9431	**0.0064**
IL-10	122 ± 44	183 ± 44	577 ± 44	0.5217	**0.0001**
IL-6	602 ± 162	932 ± 162	649 ± 162	0.2704	0.9698
TNF	1,606 ± 401	2,025 ± 401	3,353 ± 401	0.6828	**0.0083**
IL-1α	176 ± 51	117 ± 51	88 ± 51	0.6298	0.3806
IL-1β	70 ± 30	119 ± 30	67 ± 30	0.4306	0.9946
IP-10	847 ± 176	1,879 ± 176	2,423 ± 176	**0.0043**	**0.0001**
MCP-1	4,383 ± 293	4,398 ± 293	4,502 ± 293	0.9991	0.9413
MIP-1α	2,169 ± 369	2,831 ± 369	3,263 ± 369	0.3531	0.0804
MIP-1β	545 ± 229	707 ± 229	937 ± 229	0.8369	0.3852
IL36Ra	305 ± 39	308 ± 39	297 ± 39	0.9982	0.9832
VEGF	124 ± 15	114 ± 15	129 ± 15	0.8464	0.963
INFECTED					
Cytokines					
IFN-γ	7 ± 171	1,935 ± 171	1,190 ± 171	**0.0001**	**0.0001**
IL-17	7 ± 170	326 ± 170	945 ± 170	0.3168	**0.0007**
IL-4	86 ± 18	100 ± 18	193 ± 18	0.7883	**0.0003**
IL-10	163 ± 97	431 ± 97	1,204 ± 97	0.1021	**0.0001**
IL-6	936 ± 436	2,975 ± 436	786 ± 436	**0.0039**	0.9577
TNF	733 ± 378	3,651 ± 378	2,660 ± 378	**0.0001**	**0.0017**
IL-1α	16 ± 44	244 ± 44	53 ± 44	**0.0014**	0.781
IL-1β	51 ± 82	719 ± 82	128 ± 82	**0.0001**	0.7337
IP-10	1,351 ± 132	3,264 ± 132	3,575 ± 132	**0.0001**	**0.0001**
MIP-1α	800 ± 365	2,603 ± 365	2,487 ± 365	**0.0024**	**0.0044**
MIP-1β	172 ± 71	716 ± 71	687 ± 71	**0.0001**	**0.0001**
MCP-1	5,420 ± 257	5,544 ± 257	5,662 ± 257	0.9189	0.7323
IL-36Ra	255 ± 26	280 ± 26	259 ± 26	0.7254	0.9892
VEGF	224 ± 24	140 ± 24	146 ± 24	**0.0336**	0.0502
AFFECTED					
Cytokine					
IFN-γ	2 ± 289	2,895 ± 289	789 ± 294	**0.0001**	0.1132
IL-17	3 ± 74	361 ± 74	529 ± 74	**0.0037**	**0.0001**
IL-4	105 ± 19	121 ± 19	213 ± 19	0.7807	**0.0008**
IL-10	134 ± 63	374 ± 63	803 ± 63	**0.0228**	**0.0001**
IL-6	704 ± 359	3,132 ± 359	431 ± 359	**0.0001**	0.813
TNF	1,770 ± 679	8,366 ± 679	3,676 ± 679	**0.0001**	0.1009
IL-1α	81 ± 53	296 ± 53	116 ± 53	**0.0138**	0.8591
IL-1β	20 ± 48	432 ± 48	118 ± 48	**0.0001**	0.2614
IP-10	1,358 ± 218	2,832 ± 218	2,728 ± 218	**0.0001**	**0.0002**
MIP-1α	2,404 ± 892	5,594 ± 892	4,044 ± 892	**0.0315**	0.3366
MIP-1β	319 ± 71	969 ± 71	718 ± 71	**0.0001**	**0.0008**
MCP-1	4,637 ± 135	4,573 ± 135	4,467 ± 135	0.9202	0.5813
IL-36Ra	341 ± 98	347 ± 98	331 ± 98	0.9982	0.996
VEGF	154 ± 15	105 ± 15	109 ± 15	**0.0493**	0.0716

Whole blood was stimulated with PBS (nil control antigen), PPD-B and PWM (vitality control). Levels of 14 cytokines were determined through ELISA. LSM (Least Squares Mean) and SEE (Standard Estimated Error) values and statistical differences between conditions (*p*-value) are presented.

*p-value < 0.05 were considered statistically significant and are marked in bold.*

At the moment, however, we have not set a cut-off for this type of approach to define the PWM response as efficient.

In particular, the difference (∆_cytokine) between the level of each specific cytokine measured in the PWM condition and its baseline cytokine concentration (PBS) in the animals uninfected, infected and affected were statistically significant (*p* < 0.05) for 6 interleukins and chemokines (IFN-γ, IL-17, IL-4, IL-10, TNF, and IP-10). In the three groups the results were always highly statistically significant (*p* < 0.001) for IL-17, IL-10, IP-10 (Table 1).

In the uninfected group, no statistically significant differences between PPD-B and PBS conditions were observed for all cytokines, except for IP-10 (Table 1). On the contrary, in both the infected and affected group, higher levels and statistically significant (*p* < 0.05) difference (∆_cytokine) of several cytokines were detected after PPD-B stimulation compared to the baseline control (PBS): IFN-γ, IL-6, IL-1α, IL-1β, MIP-1α, MIP-1β, IP-10, TNF (Table 1). Interestingly, in both groups lower levels of VEGF-A were detected in the PPD-B condition compared to PBS. In the affected group only, we observed a higher release of IL-17 and IL-10 in response to stimulation with PPD-B.

**Figure 1:**
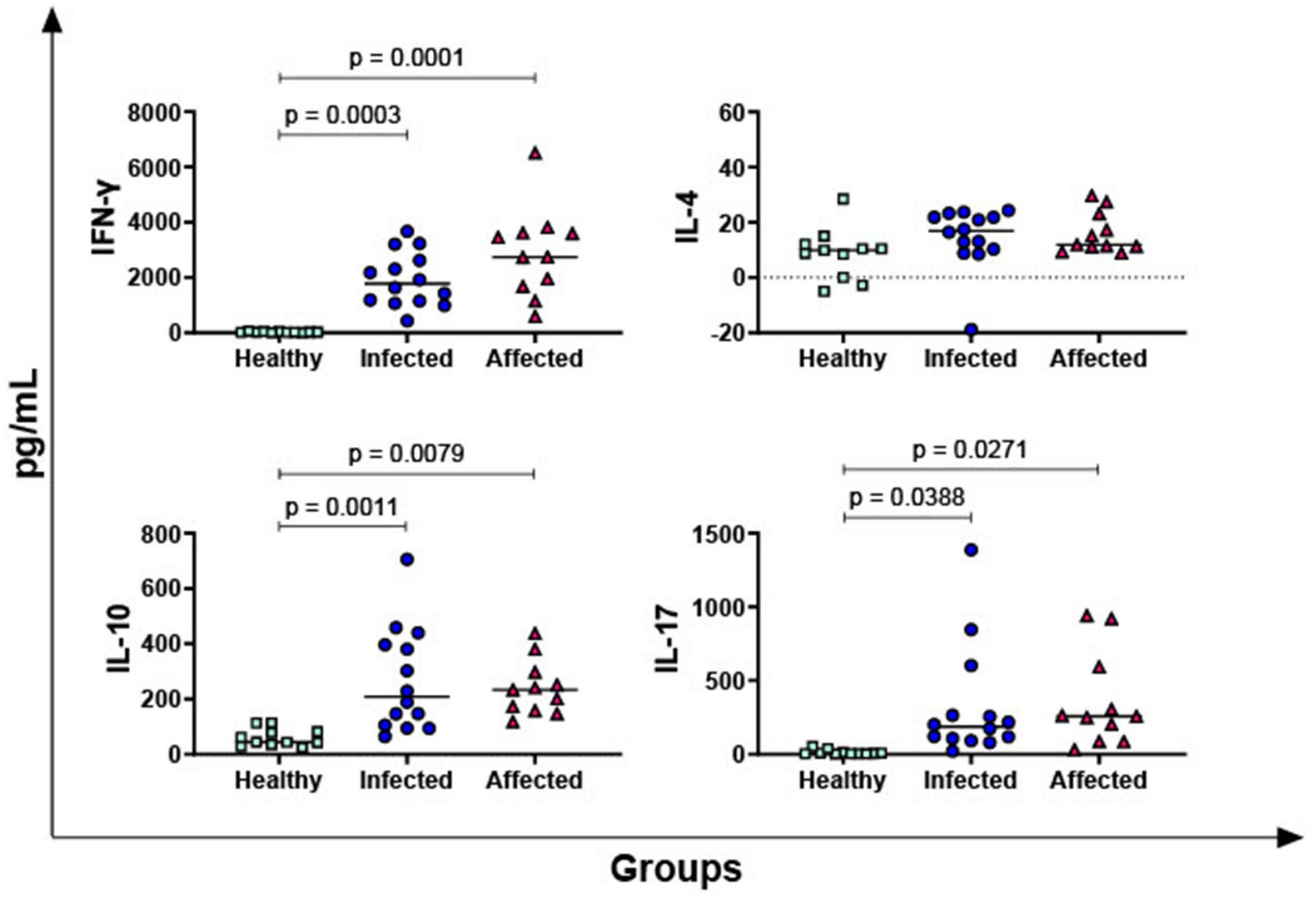
Release of *M. bovis* specific T-cell cytokines, IFN-γ, IL-4, IL-10, IL-17, in healthy, infected and affected Mediterranean buffaloes. Heparin blood from healthy (*n* = 11), infected (*n* = 14), affected (*N* = 11) animals were collected. Whole blood was stimulated with PPD-B, alongside PBS (nil control antigen). After 18–24 h, plasma were collected, and levels of IFN-γ, IL-4, IL-10, IL-17 were determined through ELISA. *Mycobacterium bovis* specific cytokines responses were determined by subtracting PBS cytokines levels from those measured in the PPD-B condition. Differences between groups are displayed and *p* < 0.05 were considered statistically significant.

Then, differences between the three groups (healthy, infected, affected) were investigated. *Mycobacterium bovis* specific cytokines responses were determined by subtracting baseline cytokines levels (PBS, nil control) from those measured in the PPD-B condition. We considered statistically significant (*p* < 0.05) the difference (∆_cytokine) between the level of each specific cytokine measured in the PPD-B condition and its baseline cytokine concentration (PBS). Affected and infected animals released higher levels of IFN-γ (2,892 ± 325; 1,928 ± 288, respectively) in response to PPD-B compared to healthy subjects (23 ± 325) with *p* < 0.0003 (Figure 1), while no significant difference was found between affected and infected group (*p* = 0.083) (Figure 1). Three other key T cell cytokines were tested: IL-4 (hallmark of Th2 response), IL-17 (mainly released by Th17), and IL-10 (an immunosuppressive cytokine released by Tregs). No significant differences in IL-4 levels among groups were observed, whereas affected and infected animals released higher levels of IL-17 and IL-10 in response to PPD-B than healthy subjects (Figure 1).

**Figure 2:**
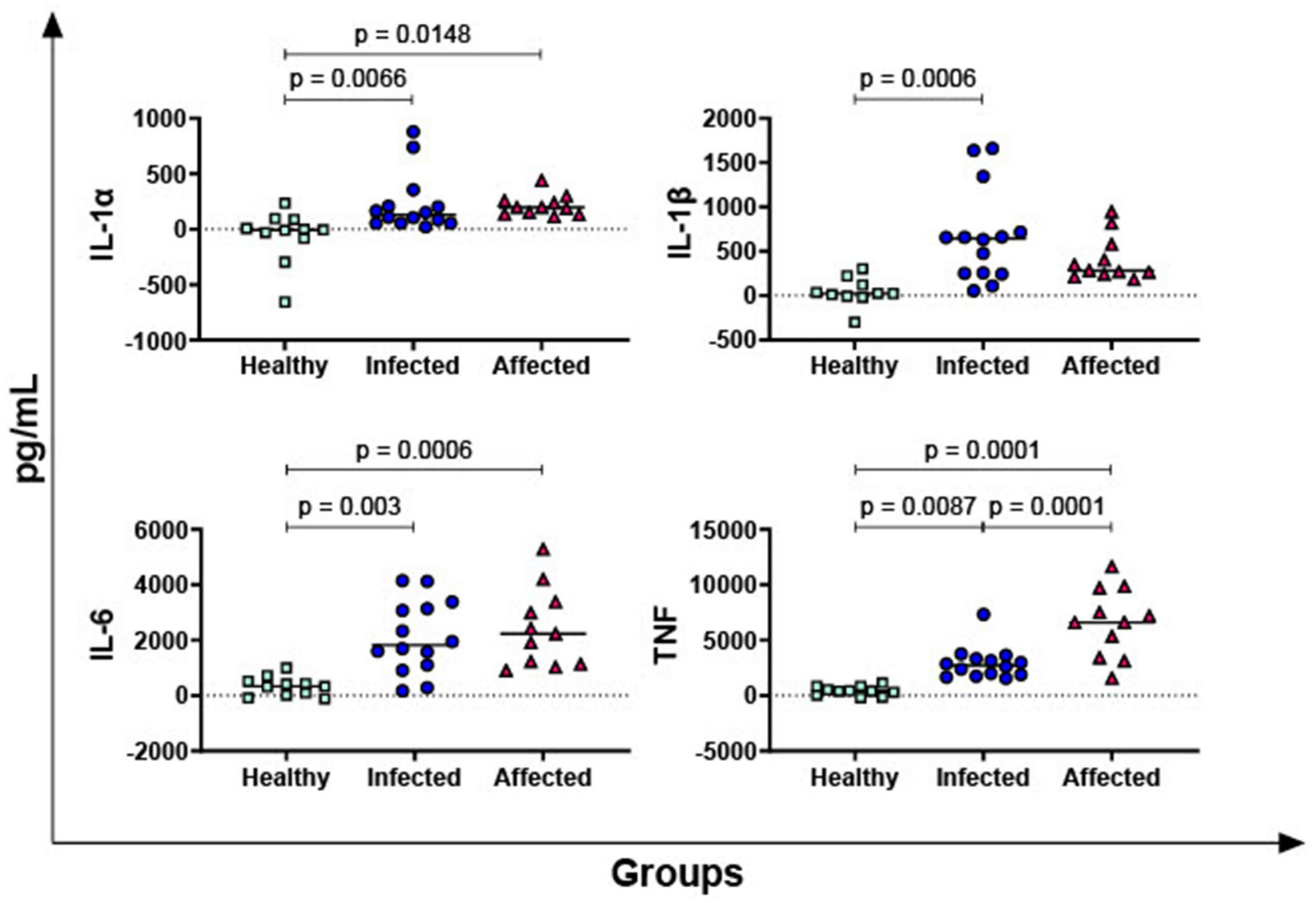
Release of *M. bovis* specific pro-inflammatory cytokines IL-1α, IL-1β, IL-6, and TNF in healthy, infected and affected Mediterranean buffaloes. Heparin blood from healthy (*n* = 11), infected (*n* = 14), affected (*N* = 11) animals were collected. Whole blood was stimulated with PPD-B, alongside PBS (nil control antigen). After 18–24 h, plasma were collected, and levels of IL-1α, IL-1β, IL-6, TNF were determined through ELISA. *Mycobacterium bovis* specific cytokines responses were determined by subtracting PBS cytokines levels from those measured in the PPD-B condition. Differences between groups are displayed and *p* < 0.05 were considered statistically significant.

IL-1α, IL-1β, IL-6, and TNF are the most important pro-inflammatory cytokines of the innate immune response (25) and their PPD-B-specific release was then investigated. As displayed in Figure 2, our data revealed that both infected and affected animals released higher levels of IL-1α, IL-6, and TNF in response to PPD-B compared to healthy subjects. In addition, *M. bovis* infected animals released higher levels of PPD-B-specific IL-1β compared to healthy animals (*p* = 0.0006) (Figure 2). Affected animals exhibited as well higher levels of PPD-B-specific IL-1β compared to healthy subjects, even if not significant (only a tendency was observed, *p* = 0.0691) (Figure 2). Moreover, affected buffaloes presented higher levels of TNF compared to animals belonging to the infected group (Figure 2).

**Figure 3:**
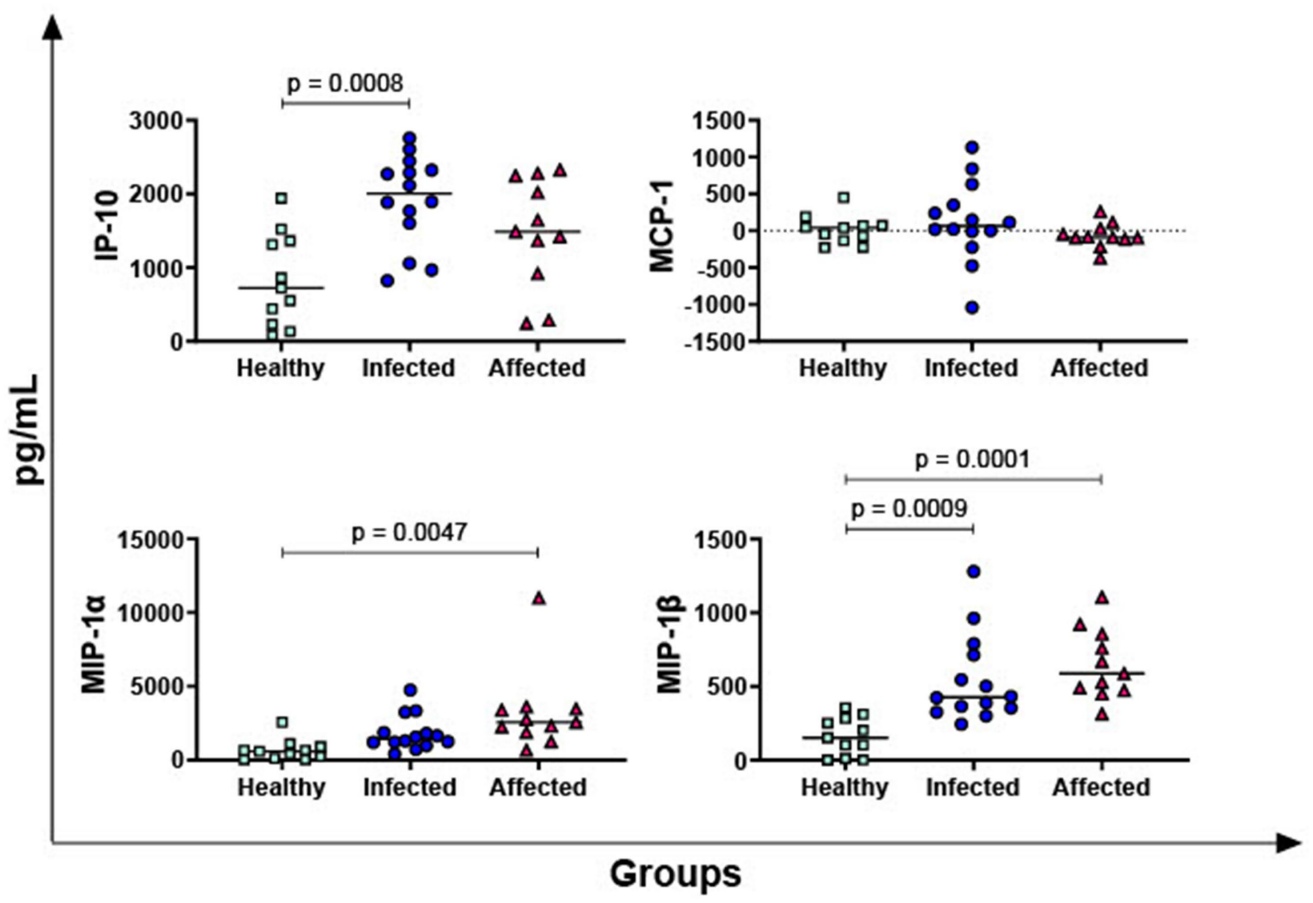
Release of *M. bovis* specific chemokines IP-10, MIP-1α, MIP-1β, MCP-1 in healthy, infected and affected Mediterranean buffaloes. Heparin blood from healthy (*n* = 11), infected (*n* = 14), affected (*N* = 11) animals were collected. Whole blood was stimulated with PPD-B, alongside PBS (nil control antigen). After 18–24 h, plasma were collected, and levels of IP-10, MIP-1α, MIP-1β, MCP-1 were determined through ELISA. *Mycobacterium bovis* specific cytokines responses were determined by subtracting PBS cytokines levels from those measured in the PPD-B condition. Differences between groups are displayed and *p* < 0.05 were considered statistically significant.

In Figure 3, the PPD-B-specific release of four key chemokines was then evaluated: IP-10, MIP-1α, MIP-1β, and MCP-1. No differences between groups were observed in terms of MCP-1, whereas both infected and affected animals released higher levels of MIP-1β in response to PPD-B compared to healthy subjects. Our data revealed that affected animals, but not infected buffaloes, released higher levels of MIP-1α in response to PPD-B compared to healthy subjects. Infected animals released also higher levels of PPD-B specific IP-10 compared to healthy subjects, whereas a tendency was noted between affected and healthy animals (*p* = 0.0711).

**Figure 4:**
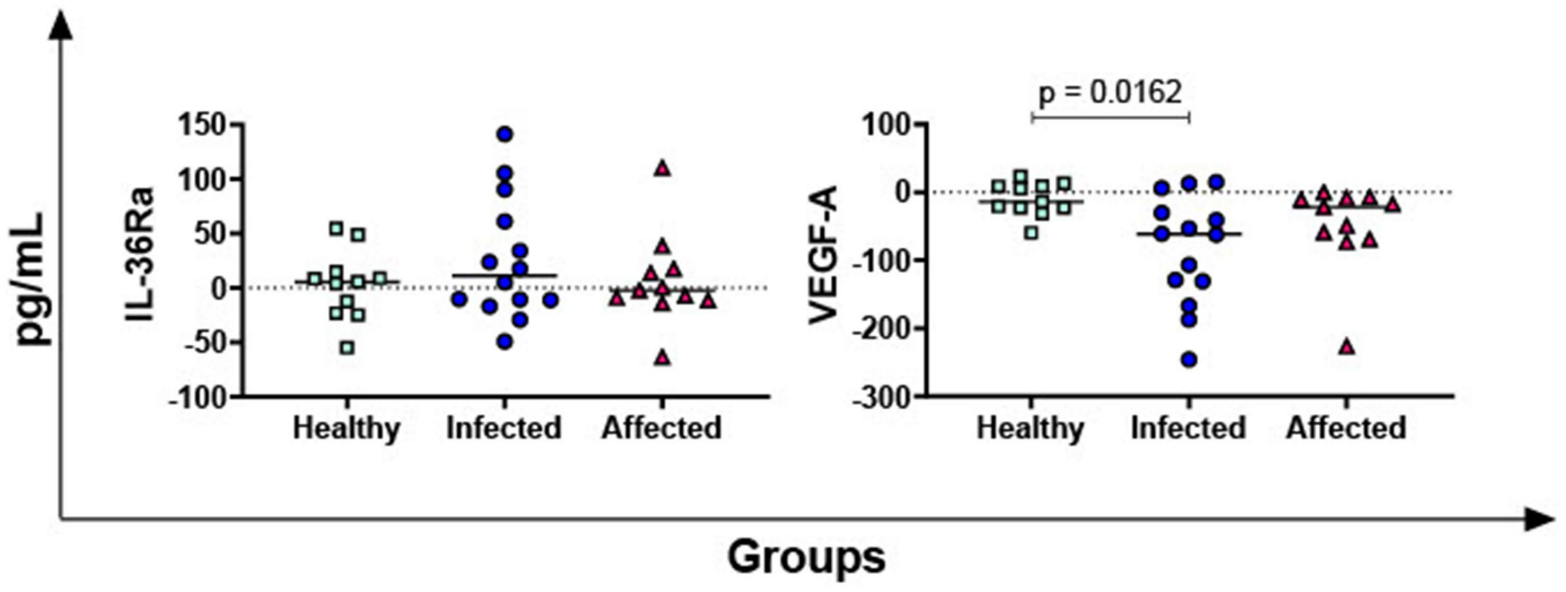
*M. bovis* specific release of IL-36Ra and VEGF-A in healthy, infected and affected Mediterranean buffaloes. Heparin blood from healthy (*n* = 11), infected (*n* = 14), affected (*N* = 11) animals were collected. Whole blood was stimulated with PPD-B, alongside PBS (nil control antigen). After 18–24 h, plasma were collected, and levels of IL-36Ra, VEGF-A were determined through ELISA. *Mycobacterium bovis* specific cytokines responses were determined by subtracting PBS cytokines levels from those measured in the PPD-B condition. Differences between groups are displayed and *p* < 0.05 were considered statistically significant.

The levels of the receptor antagonist IL-36Ra and the growth factor VEGF-A were also evaluated. No differences in IL-36Ra among groups were observed, but statistically lower PPD-B-specific release of VEGF-A was reported in the infected group compared to healthy subjects (*p* = 0.0162) (Figure 4).

**Figure 5:**
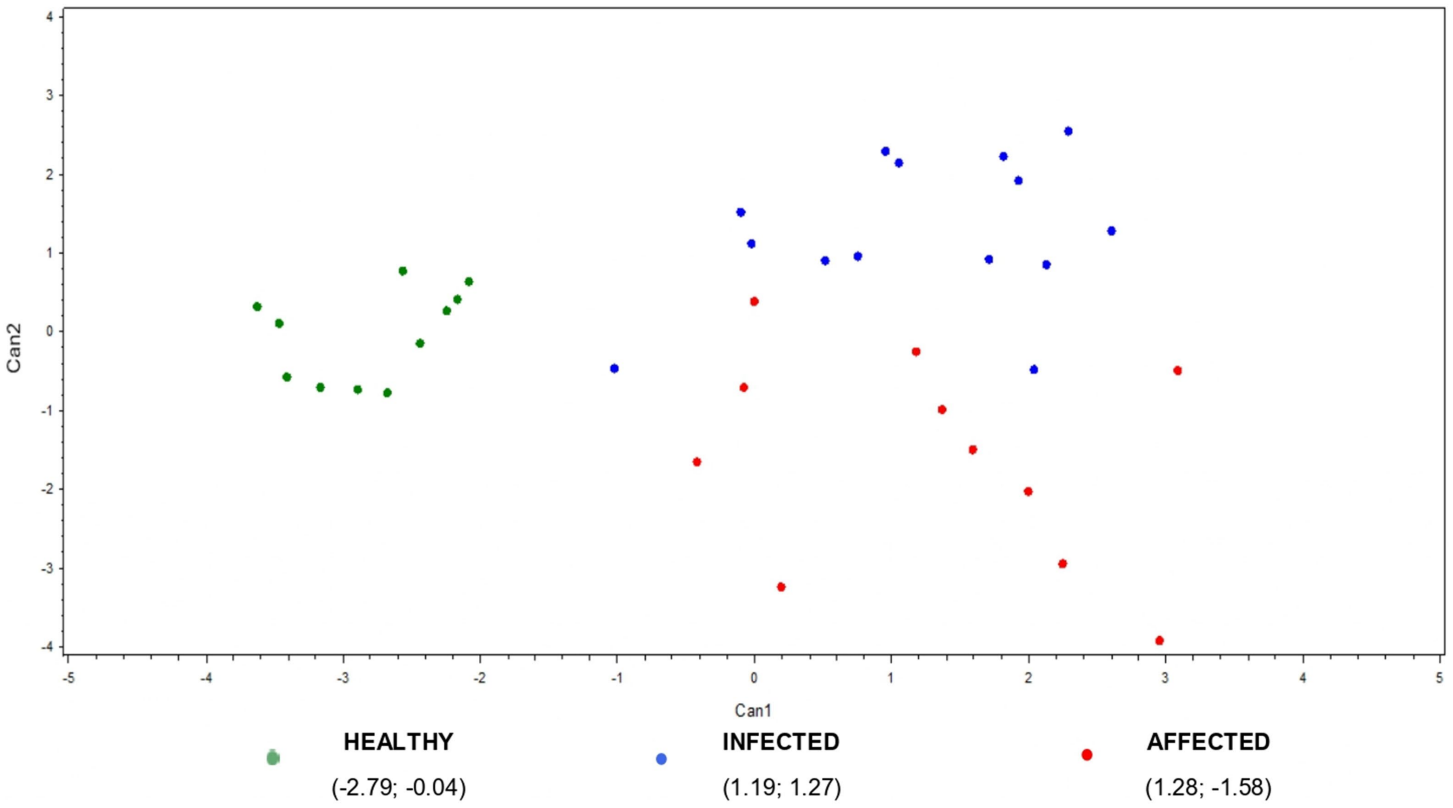
Plot from canonical discriminant analysis. A multivariate approach was conducted using canonical discriminant analysis on 11 ∆_cytokines by the CANDISC Procedure. Animals belonging to the three groups (healthy, infected, affected) are displayed based on the two canonical functions (Can1, Can2).

Finally, canonical discriminant analysis (CDA) was used to generate predictive cytokine profiles by group, in order to identify potential diagnostic biomarkers. IL-4, IL-36 and MCP-1 did not show statistically significant difference among the groups, so they were removed from the analysis. The two new functions cumulatively explained 100% of the total variance: Canonical 1 (Can1) explains 71% of the variation among the three groups with a highly significant probability (*p* < 0.0001), while canonical 2 (Can2) explains the remaining 29% with a significant probability (*p* < 0.005). The multivariate analysis results are shown in Table 2 and Figure 5. Table 2 reports the factor loading (FL) for each variable in the two canonicals, highlighting the variable weight in Can1 and Can2. The first one was positively and highly correlated with ∆_IFN-γ, ∆_IL-6, ∆_TNF, ∆_IL1-α, ∆_IL-10, and ∆_MIP1-β (FL ≥ 0.60), even if the other ∆_cytokine FL were however quite relevant. The second canonical had a strong negative correlation only with ∆_TNF (−0.62), while ∆_IL-17, ∆_IL-6, ∆_IL1-α, ∆_IL-10, and ∆_MIP-1β FL were negligible to discriminate infected from affected animals. The second canonical had also a weak negative correlation with ∆_MIP-1α and ∆_IFN-γ (−0.37; −0.31 respectively), and a slightly positive correlation with ∆_IP-10 (0.32), and ∆_IL1-β (0.33) which prevents it differentiating animals presenting or not visible TB post-mortem lesions (affected vs. infected).

**Table 2:** Total canonical structure: correlations between canonicals and original variables.

Total canonical structure		
∆_cytokine	Can1	Can2
∆_IFN-γ	**0.79**	−0.31
∆_IL-17	0.52	−0.05
∆_IP-10	0.59	0.32
∆_IL-6	**0.68**	−0.13
∆_TNF	**0.71**	**−0.62**
∆_IL-1α	**0.60**	0.04
∆_IL-1β	0.59	0.33
∆_IL-10	**0.65**	0.11
∆_MIP-1α	0.48	−0.37
∆_MIP-1β	**0.74**	−0.17
∆_VEGF	−0.45	−0.29

The difference (∆_cytokine) between the level of each specific cytokine measured in the PPD-B condition and its baseline cytokine concentration (PBS) showed their weight in the canonicals.

The heavier correlation coefficients are marked in bold.

The scatter plot of multivariate outcomes is presented in Figure 5. It shows the clear discrimination between the healthy and the infected/affected animals based on the first canonical, while the second one allows for a quite good separation between animals presenting or not visible TB post-mortem lesions (affected vs. infected animals) (Figure 5).

## 4 Discussion

Bovine tuberculosis (TB) is a worldwide zoonosis that affects many species of domestic and wild animals, including Mediterranean buffalo. This disease represents a threat to human health, and it is a great concern for buffalo producers in Italy, thus several efforts have been made over the last 10 years to eradicate it (5). An early and accurate detection of *M. bovis* infection in this specie is required for an efficient control of the disease. In the past years, several cytokine biomarkers have become a focus for diagnostic tests in human, livestock and wildlife (13). IFN-γ is a type II interferon which triggers inflammation, and promotes NK cell activity and macrophage classical polarization; its secretion is regarded as a hallmark of NK/cytotoxic T cells activation (11, 26). Studies in cattle and buffaloes reported that IFN-γ can be a useful biomarker of *M. bovis* infection (6, 13). Accordingly, our data revealed that both infected and affected buffaloes release high levels of IFN-γ in response to PPD-B stimulation compared to uninfected animals.

In our study, the release of other key immune cytokines in response to bovine purified protein derivative (PPD-B) stimulation was investigated. The simultaneous detection of numerous cytokines can indeed provide a better understanding of the cellular immune response against the intracellular pathogen *M. bovis*. A recent study in African buffaloes described the use of the multiplex microbead-based Luminex® technology to identify novel biomarkers of *M. bovis* infection, which enables the simultaneous assessment of multiple cytokines and/or chemokines (27). Using the same technology, we screened for the first time other the 13 cytokines/chemokines in Mediterreanean buffalo (27). A control of lymphocyte reactivity, PWM, was included in our experiment. All 36 animals analyzed released IFN-γ and other T cell-related cytokines (IL-4, IL-10, IL-17, TNF) in response to PWM stimulation, indicating the absence of any treatments that could have affected the analyses (e.g., corticosteroids), or immunosuppression due to pathological events, or inadequate preservation of the sample.

First, the release of IL-17 was monitored. This interleukin is mainly released by the Th subsets Th17 (28), but also γδ T-cell can be its important source. IL-17 promotes inflammation and neutrophil recruitment and activation (29). Our data revealed that both infected and affected buffaloes release high levels of PPD-B-specific IL-17 compared to uninfected animals. These data are consistent with findings in cattle, where researchers observed PPD-B-specific IL-17 release in *M. bovis* infected animals, mainly by CD4+ and γδ T cells (30, 31). It was suggested that early release of IL-17 promotes immunity to this intracellular pathogen (32), nevertheless excessive IL-17 responses may be also be detrimental (30–33). IL-17 expression was indeed associated to the presence of gross tuberculous lesions in *M. bovis*-infected cattle (33). A more recent study in African buffaloes reported that *M. bovis* infected animals release high levels of IL-17 in response to the TB antigen compared to the Nil control (27). Therefore, taking into account previous studies conducted in cattle and African buffalo and our results, it seems that IL-17 can be considered in the evaluation of the response to *M. bovis* infection also in Mediterranean buffalo.

IL-4 is an interleukin critical for the induction and perpetuation of the Th2 response, mainly characterized by antibody production (25). In all tested animals, we did not observe release in response to PPD-B stimulation and no differences were detected between groups, in agreement with previous data on African Buffaloes (27).

IL-10 is a potent anti-inflammatory and immune-suppressive cytokine, release to prevent the development of inflammatory and autoimmune pathologies (34). It is released by several cell types, including macrophages, B cells, NK cells and T cell subsets, especially Treg cells (25, 34). Our data revealed that both *M. bovis* infected and bTB affected buffaloes release high levels of PPD-B-specific IL-10 compared to uninfected controls. Contrasting results in this cytokine were found in the literature. A study reported that *M. bovis* infected African buffaloes did not release IL-10 in response to stimulation with TB antigens (27), whereas studies in goat and cattle revealed that this cytokine can promote survival of *M. bovis* in macrophages (35). IL-10 plays also an important role in granuloma formation; its expression raised in TB lesions in advanced stages of the disease (36, 37). In our study, no significant differences were observed between animals presenting or not visible TB lesions at the slaughterhouse, nevertheless, further studies on a larger set of samples should be carried out to see whether IL-10 can differentiate animals in a different stage of infection.

Subsequently, we monitored the release of four pro-inflammatory cytokines in response to PPD-B stimulation: IL-1α, IL-1β, IL-6, and TNF. These cytokines not only trigger inflammation but are also potent pyrogens and promote the synthesis of acute phase proteins from the liver during inflammation (25).

IL-1α and IL-1β are both members of the IL-1 superfamily and are released at the early stages of infection. These interleukins can enhance the release of several chemokines, with subsequent infiltration of immune cells in the inflamed tissue (38). Our data revealed that *M. bovis* infected buffaloes release high levels of PPD-B-specific IL-1α and IL-1β compared to uninfected controls. Affected animals release as well high levels of PPD-B-specific IL-1α and IL-1β compared to uninfected animals, although for the latter cytokine only a tendency was observed. Our data are in agreement with what was observed in cattle: previous studies reported that *M. bovis* infected cattle released higher levels the IL-1β in response to stimulation with TB antigens compared to uninfected animals (39, 40). In addition, Jones and colleagues observed that parallel measurement of IL-1β and IFN-γ enhanced test sensitivity for the detection of *M. bovis* infection in cattle (40).

IL-6 is a pleiotropic cytokine: it can exert both pro-inflammatory and anti-inflammatory properties. In the course of infection, this interleukin triggers the recruitment of monocytes to the inflammation site and the maintenance of Th17 cells (41). Very few studies investigated its role as a biomarker of *M. bovis* infection in cattle or other animals. In a recent study in African buffalo researchers observed that *M. bovis* infected animals release high levels of this interleukin in response to the TB antigen compared to the Nil control (27). In agreement, our data revealed that both infected and affected buffaloes release high levels of PPD-B-specific IL-6 compared to uninfected controls.

TNF is a member of the TNF superfamily and triggers as well the release of several cytokines and chemokines, which recruit leukocytes into the inflammatory site (42). In our study, we observed that both infected and affected buffaloes released high levels of PPD-B-specific TNF compared to uninfected controls. In addition, higher levels of this pro-inflammatory cytokine was observed in animals with visible TB lesions at the slaughterhouse compared to those only reactive to the IFN-γ assay. Very few studies investigated TNF role as a biomarker of *M. bovis* infection in ruminants other than cattle. In a recent study in African buffaloes it was observed that *M. bovis* infected animals released high levels of this TNF in response to the TB antigen compared to the Nil control, although differences were not statistically significant (27). Experimental studies in mice (experimental model of TB) reported that this cytokine is required for granuloma formation (43–45) and studies in humans showed that it plays a crucial role in controlling latent tuberculosis (46, 47). Our data suggest that this cytokine plays an important role in *M. bovis* infection and also in Mediterranean buffalo. It can potentially be used to identify animals in advanced stages of the disease (with TB lesions), considering that it shows a relevant correlation with Can2, the canonical function that can discriminate infected from affected animals in our study.

Subsequently, analyses were extended to four key immune chemokines. Chemokines are a group of small proteins that trigger the selective recruitment of specific cell types in inflamed tissues (48).

IP-10, also called CXCL10, is secreted by different cell types (especially antigen presenting cells) and it promotes the recruitment of monocytes, macrophages, NK cells, activated T cell to the site of inflammation (49, 50). IP-10 production is strongly enhanced by IFN-γ (49) and it plays an important role in the cell mediated immune response against Mycobacterium infection, as well as in granuloma formation (51, 52). In humans, researchers observed that IP-10 could be a potential biomarker for the diagnosis of TB (51, 53) and studies in cattle and African buffaloes showed the utility of this chemokine in identifying *M. bovis* infected animals (14, 15, 27). In agreement, our data revealed that *M. bovis* exposed Mediterranean buffaloes released higher levels of PPD-B-specific IP-10 compared to controls, suggesting that this cytokine could be as promising biomarker of TB in buffaloes. In humans, this chemokine showed promising results also in the diagnosis of latent tuberculosis infection: researchers observed a stronger IP-10 response to stimulation with TB antigens in subjects with latent TB compared to those with active TB (53). Accordingly, we observed higher PPD-B-specific release of IP-10 in infected buffaloes compared to those with visible TB lesions at the slaughterhouse, although differences were not statistically significant. In addition, it shows only a weak correlation with Can2. In the future, a higher number of animals should be screened to understand whether this chemokine can be valuable in discriminating animals in an early stage of infection (no lesions) from animals in a later stage of infection (presence of visible lesions).

MIP-1α (also known as CCL3) and MIP-1β (also named CCL4) are chemokines mainly produced by monocyte/macrophages (54) and possess several inflammatory properties, such as chemotaxis of monocytes, dendritic cells, T cells, NK cells, and granulocytes (55). Both cytokines are involved in granuloma formation during mycobacterial infection, promoting cells recruitment to the site of mycobacterium infection (52). Our data showed that all affected animals release high levels of both MIP-1α and MIP-1β in response to PPD-B-specific stimulation compared to healthy subjects. These data are in agreement with what was observed in African buffaloes (27) and suggest that these chemokines could be a useful biomarker of TB in Mediterranean buffaloes as well. Slightly different results were observed in the infected group. Our data showed that infected animals release high levels of MIP-1β, but not MIP-1α, in response to PPD-B-specific stimulation compared to the healthy group. This might suggest that MIP-1α might be useful to differentiate animals in the early stage of infection from those in the later stage of infection (presence of visible lesions). In fact, Δ_MIP-1α has the second value most important in Can2 even if it might be considered a weak correlation.

MCP-1, also called CCL2, is a potent chemoattractant for monocytes, but it can promote migration of other cell types (e.g., T cells, NK cells and dendritic cells) to the inflammatory site (*56*). Monocytes and macrophages are its main source (*56*). Previous studies in mice reported that this cytokine is important in granuloma formation, promoting monocyte and macrophage recruitment to the site of mycobacterium infection (*52*). Despite that, in our work no differences between groups were observed in terms of MCP-1.

Then, we monitored the PPD-B-specific release of a receptor antagonist: IL-36Ra. Receptor antagonists play a crucial role in controlling inflammation, in order to prevent potentially pathological over-response to stressors (38). IL-36Ra is the receptor antagonist of another IL-1 family member: the pro-inflammatory IL-36. No differences between PPD-B-treated and PBS samples were observed in all tested animals. In addition, no differences were observed between groups. These data are in agreement with previous studies in African Buffalo, where both IL-36Ra and IL-1Ra (another the receptor antagonist belonging to the IL-1 family) were not released in response to stimulation with TB antigens (27, 57).

Finally, the PPD-B-specific release of the vascular growth factor A was monitored. VEGFs are crucial in the physiological development and maintenance of the vascular and lymphatic systems. VEGF-A promote endothelial cell survival and angiogenesis (58). Previous studies reported that this growth factor plays an important role in granuloma formation during mycobacterial infection (59). In details, a study in mice showed that mycobacterial granulomas contain a subpopulation of VEGF-A-producing macrophages, and this cytokine promoted macrophage recruitment to the granuloma via a non-angiogenic pathway (59). In our study, we observe no release of VEGF-A in response to PPD-B-stimulation in all tested Mediterranean buffaloes. In addition, lower PPD-B-specific release of VEGF-A was observed in the infected group compared to healthy animals. A recent study in African Buffaloes reported as well that *M. bovis* infected animals did not release this growth factor in response to TB antigen (27).

In addition, CDA allows a generation of a predictive cytokine/chemokine set, that can potentially be used to discriminate healthy from *M. bovis* exposed animals (infected/affected groups). Quantitative determination of the chemokine MIP-1β, the anti-inflammatory IL-10, and three pro-inflammatory cytokines (IL-6, IL-1α, and TNF) performed in parallel with the well-known IFN-γ test could improve the sensitivity of the TB diagnoses in buffalo. Moreover, ∆_TNF can be valuable in distinguishing between *M. bovis*-exposed animals in various stages of infection: infected (no TB-like post-mortem lesions) and affected (visible TB-like post-mortem lesions). Additionally, despite their limited contribution to the differentiation between the two groups, ∆_MIP-1α, ∆_IFN-γ, ∆_IP-10, and ∆_IL1-β can be considered promising biomarkers for the detection of TB in buffaloes.

Our data suggest that some cytokines could be useful biomarkers of TB in Mediterranean Buffalo. These preliminary observation should be validated on a larger set of samples, in order to properly establish the sensitivity and specificity of these ELISAs. As performed in our previous study (6), samples from buffaloes infected with other mycobacteria should be included in the analysis, in order to evaluated the specificity of these potential biomarkers.

In conclusion, in this work we observed that IL-10, TNF, IL-1α, IL-6, MIP-1β could be useful biomarkers of TB in Mediterranean Buffalo and parallel measurement of some of these most promising cytokines will likely improve the diagnosis of *M. bovis* infection in this specie.

## Data Availability

The raw data supporting the conclusions of this article will be made available by the authors without undue reservation.
